# Specialized Pro-resolving Lipid Mediators: Modulation of Diabetes-Associated Cardio-, Reno-, and Retino-Vascular Complications

**DOI:** 10.3389/fphar.2018.01488

**Published:** 2018-12-19

**Authors:** Monica de Gaetano, Caitriona McEvoy, Darrell Andrews, Antonino Cacace, Jonathan Hunter, Eoin Brennan, Catherine Godson

**Affiliations:** ^1^UCD Diabetes Complications Research Centre, Conway Institute and UCD School of Medicine, University College Dublin, Dublin, Ireland; ^2^Renal Transplant Program, University Health Network, Toronto, ON, Canada

**Keywords:** diabetic kidney disease, diabetes-associated atherosclerosis, diabetic retinopathy, lipoxins, resolvins

## Abstract

Diabetes and its associated chronic complications present a healthcare challenge on a global scale. Despite improvements in the management of chronic complications of the micro-/macro-vasculature, their growing prevalence and incidence highlights the scale of the problem. It is currently estimated that diabetes affects 425 million people globally and it is anticipated that this figure will rise by 2025 to 700 million people. The vascular complications of diabetes including diabetes-associated atherosclerosis and kidney disease present a particular challenge. Diabetes is the leading cause of end stage renal disease, reflecting fibrosis leading to organ failure. Moreover, diabetes associated states of inflammation, neo-vascularization, apoptosis and hypercoagulability contribute to also exacerbate atherosclerosis, from the metabolic syndrome to advanced disease, plaque rupture and coronary thrombosis. Current therapeutic interventions focus on regulating blood glucose, glomerular and peripheral hypertension and can at best slow the progression of diabetes complications. Recently advanced knowledge of the pathogenesis underlying diabetes and associated complications revealed common mechanisms, including the inflammatory response, insulin resistance and hyperglycemia. The major role that inflammation plays in many chronic diseases has led to the development of new strategies aiming to promote the restoration of homeostasis through the “resolution of inflammation.” These strategies aim to mimic the spontaneous activities of the ‘specialized pro-resolving mediators’ (SPMs), including endogenous molecules and their synthetic mimetics. This review aims to discuss the effect of SPMs [with particular attention to lipoxins (LXs) and resolvins (Rvs)] on inflammatory responses in a series of experimental models, as well as evidence from human studies, in the context of cardio- and reno-vascular diabetic complications, with a brief mention to diabetic retinopathy (DR). These data collectively support the hypothesis that endogenously generated SPMs or synthetic mimetics of their activities may represent lead molecules in a new discipline, namely the ‘resolution pharmacology,’ offering hope for new therapeutic strategies to prevent and treat, specifically, diabetes-associated atherosclerosis, nephropathy and retinopathy.

## Introduction

Diabetes and its associated complications pose a challenge to human health on a global scale. It is estimated that 425 m people are currently living with diabetes and this is predicted to rise to 700 m by 2025 The Lancet, 2018. The rapid rise in diabetes and its associated complications over the past three decades reflects numerous factors including aging, obesity, urbanization and greater longevity amongst patients (Zimmet et al., 2001). Among NCCDs, diabetes is one of the major global causes of premature mortality. It is frequently underestimated because very often persons with diabetes die from causes related to co-morbidities (Lee I.M. et al., 2012).

The prevalence of diabetes in adults worldwide is predicted to be higher in developed than in developing countries, while, the incidence of diabetes is predicted to be higher in developing countries (Wild et al., 2004). Thus, by the year 2030, the countries with the largest number of people with diabetes are predicted to be India, China and the United States; the countries with increased prevalence of overweight and obese inhabitants, the main drivers of T2D (Yach et al., 2006). The burden on already challenged health care systems is unprecedented.

Diabetes is essentially a disorder of glucose homeostasis. Conventionally, diabetes has been classified as Type-1, Type-2 and gestational diabetes. In T1D, autoimmune destruction of the β-cells of the pancreas creates an insulin-deficient state where patients are dependent on exogenous insulin for survival. The precise mechanisms underlying the pathogenesis remain elusive, but it is likely that genetic and environmental factors converge to drive an autoimmune response. The observed increasing incidence of T1D in developed nations is thought to reflect responses to environmental triggers. Historically, T1D is considered to represent 10% of total number of persons with diabetes (You and Henneberg, 2016). In T2D, peripheral insulin resistance in target tissues (including skeletal muscle, adipose tissue and liver coupled with hypersecretion of insulin) typically precedes eventual β-cell loss. The diabetes epidemic is commonly attributed to T2D (Tuomi, 2005). Gestational diabetes describes insulin resistance observed during pregnancy, which generally resolves in the postnatal period. However, these mothers are at increased risk of T2D in later life (Buchanan and Xiang, 2005).

It is now clear that the above classifications are an over-simplification. It has recently been proposed to re-define diabetes based on six clinical parameters [BMI, age at diagnosis, hemoglobin A1c, glutamate decarboxylase autoantibodies (GADAs) (evidence of autoimmunity); β-cell function and insulin sensitivity]. This has led to the identification of five distinct pathologies associated with different disease progression and risks of complications. Further characterization of the genetic architecture of these subgroups may facilitate identification of patients most at risk of specific complications (Rossing, 2018). According to the proposed classification, the sub-groups can be defined as: *(1) SAID:* This is the least common subtype (6.4%) and traditionally classified as T1D. These patients had an early onset of disease and were positive for GADAs, had low BMI and were dependent on exogenous insulin (Abegunde et al., 2007). *(2) SIDD:* these defined as a group of patients who showed insulin deficiency and were GADAs negative. This group was at greatest risk of DR (Jingi et al., 2017). *(3) SIRD:* This group represented 15.3% of the whole cohort of participants. These patients had a high degree of insulin resistance and were likely to be overweight or obese and showed kidney damage more frequently than other groups. They also had a higher risk of non-alcoholic fatty liver disease (Dutta and Mukhopadhyay, 2018). *(4) MOD:* Around a fifth of all participants were classified in cluster 4. These patients typically had high BMIs but they did not show insulin resistance (Kahn et al., 2006). *(5) Mild age-related diabetes (MARD):* Most of the patients (nearly 40%) in the cohort belonged to cluster 5. They were usually older adults with healthier metabolic profiles (including lower BMIs) than the other clusters (Ma et al., 2018).

Diabetes is associated with serious life-threatening and life limiting complications. Acute complications include hyperglycemia-induced ketoacidosis and hypoglycemia. The chronic vascular complications of diabetes have a massive impact on morbidity and mortality. These are classically defined as microvascular and macrovascular complications and reflect responses of susceptible individuals to hyperglycemia, dyslipidemia and hypertension associated with diabetes (Orban et al., 2018). The macrovascular complications include accelerated-CVD and accelerated-atherosclerosis, as discussed below (Duncan et al., 2003).

Complications of the microvasculature include retinopathy, neuropathy and nephropathy. DR is major cause of blindness in the working class (Duh et al., 2017). Diabetic neuropathy develops in almost half of all individuals with diabetes and the lifetime risk of lower limb amputation as much as 15% in certain populations. Diabetic neuropathy is a syndrome encompassing both somatic and autonomic branches of the peripheral nervous system, and, furthermore, contributes to the pathology of other diabetic complications, such as impaired wound healing and erectile dysfunction (Russell and Zilliox, 2014). As will be discussed in more detail below, DKD is the leading cause of ESRD (Piccoli et al., 2015). DKD typically develops over a long period (decades) and, importantly, it is a major risk factor for the development of macrovascular complications, including MI and stroke.

With best medical care the risk of major chronic complications for T1D are cited as 47% for retinopathy, 17% for nephropathy and 14% for CVD. These figures represent a lifetime risk. Figures for T2D are more complex. Although death rates are higher for people with diabetes, relative to age and sex matched cohorts, a recent study has shown that in the United States, whereas death rates for people with and without diabetes have fallen, the greatest decline in mortality was actually seen in those with diabetes, presumably reflecting improved management of glycemia, lipids and hypertension (Gregg et al., 2018). Moreover, in a United Kingdom study, patients with T2D initiated on metformin monotherapy had longer survival than did matched, non-diabetic controls (Bannister et al., 2014). However, the overall mortality in T2D is 60% higher than non-diabetic age and sex matched controls. One consideration on these data is that the lower rates reflect the relatively recent increase in incidence. Mortality is typically associated with chronic complications, such as DKD which develops over decades. The increased incidence may represent a timebomb of diabetes-associated mortality. Indeed, among adults with diabetes, in the United States the prevalence of ESRD has shown the smallest decrease as compared to other diabetic complications (Gregg et al., 2014). To an extent this may reflect the efficacy of preventing atherosclerosis, resulting in increased survival and increased opportunity to develop complications as a consequence of chronic exposure to hyperglycemia. As discussed below, it also reflects the need for therapeutic interventions to specifically target DKD and associated-RF.

This review will focus on describing recent advances in the understanding and elucidation of the underlying mechanisms and in exploring the potential of novel therapeutic approaches for treating diabetes-accelerated atherosclerosis, kidney disease and retinopathy, by using animal and human studies. For more comprehensive reviews of diabetic complications, readers are referred to several excellent recent reviews (Forbes and Cooper, 2013; Papatheodorou et al., 2016; Lotfy et al., 2017).

## Diabetes-Associated Atherosclerosis

### Definition of DAA

Atherosclerosis is a leading cause of vascular disease worldwide and accounts for about 50% of all deaths in westernized societies and 30% in developing countries (Fuster and Kelly, 2011). Its major clinical manifestations include IHD and ischemic stroke (Lusis, 2000), being, respectively, the world’s first and third causes of death (Barquera et al., 2015).

The strong association between diabetes, low-grade inflammation and atherosclerosis, accounts for one of the major diabetes complications worldwide: DAA (Duncan et al., 2003). Approximately 50% of patients with T2D die prematurely of a cardiovascular cause, and a further 10% die of renal failure (van Dieren S et al., 2010).

Since Ross and Libby redefined atherosclerosis as a progressive, chronic, dyslipidemic and also “inflammatory” disease, advances in basic knowledge of this multifactorial disease defined a key for inflammation in mediating all the phases of athero-progression (Ross, 1999; Libby et al., 2002). Among the numerous markers of high- and low-grade inflammation, C-reactive protein predicts the risk of atherosclerotic complications (see below) (Ross, 1999; Libby et al., 2002). In the recent trial of anti-IL-1β antibody in a large population of high risk atherosclerosis patients (CANTOS), the intervention reduced inflammation and cardiovascular events. Greatest impact was seen in those with highest baseline markers of systemic inflammation. However, its efficacy was similar in those with and without diabetes and, despite decreasing inflammatory markers, did not reduce the incidence of diabetes (Weber and von Hundelshausen, 2017; Everett et al., 2018).

### Risk Factors for DAA

The main modifiable risk factors for atherosclerosis have been identified, and they include, but are not limited to, smoking, adiposity, blood pressure, high levels of BMI, high level of LDL, low level of HDL and diabetes (Herrington et al., 2016). T2D is associated with an increased risk of CVD. A role for the lipid-lowering therapy with statins for the primary prevention of CVD in diabetes was demonstrated in CARDS, the first large primary prevention study determining the action of statins in T2D patients, e.g., the efficacy of atorvastatin in preventing disease irrespective of LDL levels (Colhoun et al., 2004). Over the past two decades, developed countries have been able to reduce the contribution of the above mentioned risk factors to mortality, whereas developing countries show an increasing trend due to high BMI and glucose (Barquera et al., 2015).

More recently, the prevalence of coronary atherosclerosis was found to be higher in diabetic than in non-diabetic patients and to be similar for diabetic individuals without clinical CAD and non-diabetics with clinical CAD, implying that prevention measures for asymptomatic diabetic individuals should be similar to secondary preventive approaches among non-diabetic population, as an aggressive prevention measure for atherosclerosis in all diabetic patients, independently of their CAD symptoms (Goraya et al., 2002).

### Cellular Pathogenetic Mechanism of DAA

**FIGURE 1:**
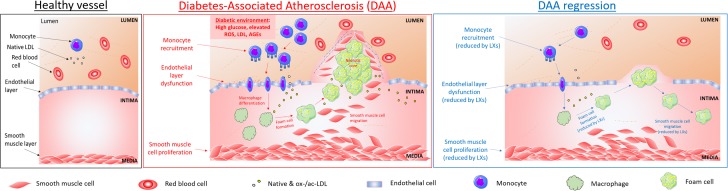
Cellular targets of SPMs in diabetes-associated atherosclerosis. DAA is typified by lesion formation due to inflammatory signals leading to monocyte recruitment into the intima, where they differentiate into macrophages and internalize native and modified lipoproteins, resulting in lipid-loaded foam cells, which accumulate in a necrotic core. Key steps in atherogenesis and progression which may be attenuated by SPMs are highlighted above and include endothelial activation, proliferation and migration (Baker et al., 2009; Brennan et al., 2018a); leukocyte (PMNs and monocytes) recruitment and activation (Colgan et al., 1993; Godson et al., 2000); foam cell transformation and necrotic core formation (Sha et al., 2015); SMCs proliferation and migration (Petri et al., 2015).

The pathogenesis of atherosclerosis shares several features with other inflammatory diseases, including the infiltration of monocytes and subsequent differentiation to macrophages in response to locally generated signals (Scrivo et al., 2011). At cellular and subcellular levels, inflammatory stimuli or a disturbed blood flow induce endothelial dysfunction (Cunningham and Gotlieb, 2005), altering the homeostatic equilibrium depicted in Figure 1 (left). This vasoreactivity allows lipoproteins apo-B to enter the intima and bind to proteoglycans which trap the LDL particles and increase their susceptibility to oxidation, acetylation and hydrolysis by secretory phospholipases thus amplifying the inflammatory response, characterized by chemokine secretion and adhesion molecules expression on ECs surface. These modifications of lipoprotein induce their aggregation in complexes and subsequent retention, and, additionally, induce monocytes recruitment, a crucial step in early phases of atherogenesis (Lusis, 2000).

Once chemoattracted to the inflammatory injury area, the monocyte undergoes a series of processes that allow cell locomotion (i.e., rolling, adhesion, polarization, crawling) to reach the endothelial transmigration sites, in proximity of low shear stressed athero-prone regions, where blood flow is disturbed, such as bifurcations of arteries (Cunningham and Gotlieb, 2005). Once transmigrated and eventually infiltrated into the intima layer, monocytes differentiate into macrophages in response to locally produced factors, such as M-CSF (Moore and Tabas, 2011). This program of differentiation includes upregulation of class A- macrophage scavenger receptor (SR-A), CD 36 (CD-36) and other cell surface receptors, to facilitate ox-LDL or ac-LDL uptake: in physiologic conditions, this process allows an efficient removal of excessive lipids from the blood circulation (“scavenging” action of macrophage). When homeostasis fails, dysregulation of this phenomenon leads to uncontrolled accumulation of lipids and cholesterol derivatives in macrophages, and their transformation into foam cells in the subintima endothelial layer (Kunjathoor et al., 2002).

Lipid-loaded foam cells, cellular debris, calcium deposits and connective tissue contribute to generate the so-called *fatty streak* (a hallmark and first sub-clinical sign of atherosclerosis), increasing inflammation and inducing necrosis and foam cell death. As the lesion grows invade both the luminal space and the intima. The necrotic area is confined within a fibrous cap made of connective tissue, composed of SMCs and collagen. Fibrous cap atheromas are the first clinically detectable atherosclerotic lesions (Li and Glass, 2002).

As the lipid core increases in size, the fibrotic cap is invaded by macrophages and lymphocytes, inducing the thinning of the cap. The mechanism by which a sustained macrophage invasion weakens the fibrous cap involves phagocytosis of the ECM and the release of proteolytic enzymes (i.e., plasminogen activators and matrix metalloproteinases, MMPs). The thinned fibrous cap is prone to rupture, exposing the inflammatory and thrombogenic molecules (TF, collagen) of the lipid core, highly increasing the risk of thrombosis (Pepine, 1998).

### Molecular Pathogenetic Mechanisms of DAA

As above stated, diabetes accelerates atherosclerosis, contributing to higher rates of mortality and morbidity among diabetic patients. The molecular mechanisms behind this likely reflects increased inflammation and decreased blood flow (Abe and Berk, 2013) but are not fully understood. Several possible triggers have been thus far hypothesized, including hyperglycemia, insulin resistance, increased activation of PDGF-dependent pathways, increased level of TF or decreased level of HDL, and AGEs and their receptors (RAGE) signaling activation (Beckman et al., 2002). Insulin and hyperglycemia play key roles in distinct phases of disease progression, *via* different mechanisms and differentially affecting the three major cell types: SMCs, macrophages and ECs (Bornfeldt and Tabas, 2011).

It has been hypothesized that, in advanced plaques, insulin resistance may promote apoptosis of PMNs, SMCs, and macrophages. In particular, death of SMCs can lead to the thinning of the fibrotic cap, whereas death of macrophages is associated with a defective phagocytic clearance of the cells (*efferocytosis*), promoting plaque necrosis (Greenlee-Wacker, 2016). These processes converge to precipitate plaque rupture and acute thrombotic vascular occlusion (Brophy et al., 2017). A relation between diabetes obese-induced adiposity and atherosclerosis in young adults have been observed (McGill et al., 1995). Elevated SFAs has been associated with obesity and insulin resistance (Funaki, 2009) and causes defective *efferocytosis* of apoptotic macrophage (Li et al., 2009), subsequently causing a secondary cellular necrosis and inflammation amplifying the plaque necrosis (Thorp and Tabas, 2009). The combined pro-apoptotic effect of macrophage insulin resistance and the anti-efferocytic effect of SFAs may create a “perfect storm” for plaque necrosis, as proposed by Bornfeldt and Tabas (2011).

Hyperglycemia accelerates formation of early/mid stage lesions of atherosclerosis by promoting an inflammatory phenotype of which adhesion molecule expression in ECs is a hallmark. Increased flux through the aldose reductase pathway accelerates glucose metabolism and generates ROS. Increased adhesion molecule expression leads to increased monocyte/macrophage accumulation and atherogenesis. In SMCs, a principal effect of increased glucose uptake appears to be increased secretion of the monocyte chemoattractant protein-1, a chemokine which acts in concert with ECs. This leads to an increased production of endothelium-derived contracting factors, which oppose the protective activity of nitric oxide (Meininger et al., 2000; vanDam et al., 2000). Ultimately, this leads to greater recruitment of monocytes into the growing lesion (Bornfeldt and Tabas, 2011), thereby further contributing to an enhanced inflammatory response. Those events have been shown to promote adventitial inflammation and *vasa vasorum* neovascularization in experimental models of diabetic atherosclerosis. In particular, over the past two decades, the work from Cosentino and Luscher (1998) has established the strong relationship between hyperglycemia, oxidative stress and inflammation, together with an increased risk of CVD in T2D (Beckman et al., 2013; Paneni et al., 2013). Very recently, their studies demonstrated epigenetic regulation of immune-metabolic pathways to increased inflammation, neovascularization and intraplaque hemorrhage in human diabetic atherosclerosis (Guzik and Cosentino, 2018).

Insulin and hyperglycemia are not the only possible factors so far correlated to the underlying pathogenetic mechanism of DAA. Hyperglycemia enhances shear stress-induced platelet activation (Gresele et al., 2003). PDGF has been shown to play a major role in the pathology of vascular diseases. Inhibition of PDGF receptor activation attenuates DAA in experimental models (Lassila et al., 2004).

The inflammatory component of microangiopathic processes is independently associated with plaque rupture, leading to coronary thrombosis. TF, the most potent trigger of the coagulation cascade, is increased in diabetic patients with poor glycemic control. Circulating TF microparticles are also associated with apoptosis of plaque macrophages, closing the link among inflammation, plaque rupture and blood thrombogenicity (Fallon et al., 1997; Singh et al., 2012).

AGE/RAGE signaling has been a well-studied cascade in many different disease states, particularly diabetes. It heavily influences both cellular and systemic responses to increase bone matrix proteins through activation of PKC, p38 MAPK, TGFβ, NFκβ and ERK1/2 signaling pathways in both hyperglycemic and calcification conditions. AGE/RAGE signaling has been shown to increase oxidative stress and to promote diabetes-mediated vascular calcification through activation of NADPH oxidase-1 and decreased expression of superoxide dismutase-1. AGE/RAGE signaling in diabetes-mediated vascular calcification is also attributed to increased oxidative stress resulting in the phenotypic switch of SMCs to osteoblast-like cells in AGEs-induced calcification (Kay et al., 2016). HDL, responsible for free cholesterol removal, are reduced in patients with insulin resistance and diabetes, conditions for which the role of obesity is highly detrimental (Rashid and Genest, 2007; Barter, 2011). In addition to their role as lipid lowering agents, *via* inhibition of 3-hydroxy-3-methylglutaryl coenzyme A reductase, pleiotropic responses to statins may include reduction of SMCs proliferation, as observed in *in vitro* and *ex vivo* models (Fleg et al., 2008).

### Current Therapies in DAA

There are currently no available therapies for the regression of atherosclerosis (Fuster et al., 1998). Therefore, new therapeutic targets are needed in order to offer an alternative type of intervention to invasive surgery, such as stenting or endarterectomy. Current therapies in DAA adopted antiplatelet/anticoagulant therapy, stabilizing the plaque (Engelberg et al., 1956; Colwell, 1997), including the use of low-dose aspirin (75–162 mg/day) for secondary prevention of cerebrovascular and cardiovascular events in all diabetic patients (Angiolillo, 2009). Evidence that LDL causes CVDs is overwhelming. It has also been proven beyond all doubt that lowering the level of LDL using statins reduces cardiovascular risk. However, many people remain at high risk even when their level of LDL has been reduced by aggressive treatment with statins. One reason for this residual risk can be a low level of HDL, an independent, inverse predictor for CAD. It has therefore been suggested that raising the level of HDL should be considered as a therapeutic strategy for reducing the residual cardiovascular risk that persists in some people, despite aggressive LDL-cholesterol lowering with statins. HDL particles have several functions with the potential to protect against arterial disease, the best known of which relates to their ability to promote cholesterol efflux from macrophages in the artery wall. However, HDLs have several additional protective properties that are independent of their involvement in cholesterol metabolism. For example, they have properties that reduce oxidation, vascular inflammation and thrombosis, improve endothelial function, promote endothelial repair, enhance insulin sensitivity and promote insulin secretion by pancreatic β islet cells (Barter, 2011). These beneficial effects may be responsible for coronary plaque stabilization in patients treated with those molecules which can up regulate HDL expression including Apo-A1 or peroxisomal proliferator-activated receptors agonists, holding great promise in the treatment of diabetic atherosclerosis.

### The Regression of DAA

The regression of existing lesions is the holy-grail in management of atherosclerosis. Over the past two decades major advances have been made to this end. Fisher’s lab and his collaborators Young, Hazen, Smith and Moore have firmly established the principle that regression of atherosclerosis is a possible therapeutic goal (Fisher, 2016).

Although monocytes are recruited into the plaque during its growth, they also have the capacity to emigrate from atherosclerotic lesion. Using murine models of regression, including the “*transplantation mouse*,” a transplant model in which plaque-bearing aortic segments are transferred into normolipidemic mice (Reis et al., 2001); the “*reversa mouse*,” a genetic “switch” model in which LDL production can be conditionally reduced in LDL-R^-/-^ mice (Feig et al., 2011); and acute treatment models, in which Apo-E^-/-^ mice are injected either with Apo-A1 (Hewing et al., 2014), with a microsomal triglyceride transfer protein inhibitor or with an anti-microRNA (miR) (anti-miR-33) (Rayner et al., 2011; Moore et al., 2013; Distel et al., 2014) a decrease in plaque size and, consequently, regression of pre-existing atherosclerosis was demonstrated (Llodra et al., 2004; Randolph, 2008; Feig et al., 2009). A possible explanation of the reduction in CD68^+^ macrophage cell content is that monocytes can enter the lymphatic system, reaching the lymph nodes, or they can migrate across the arterial endothelium toward the artery lumen to directly enter the circulating bloodstream (Llodra et al., 2004; Randolph, 2008). The main processes involved in atherogenesis are also the main target for regression, namely, the retention of apo-B-containing lipoproteins in the arterial wall and the reaction of macrophages to these particles (Williams and Tabas, 2005). The resulting foam cells secrete pro-inflammatory cytokines and chemokines, as well as retention factors that amplify the inflammatory response and promote macrophage *chemostasis*. These accumulating macrophages experience endoplasmic reticulum stress, which, if prolonged, results in apoptosis. This cell death, coupled with defective *efferocytosis*, due to an uncontrolled lipid accumulation, in which essentially SFAs decrease the fluidity of the plasma membrane, leads to the formation of the necrotic core that is characteristic of advanced plaques (Funaki, 2009; Thorp and Tabas, 2009; Bornfeldt and Tabas, 2011).

The key mechanisms that promote regression are: lipid unload of the foam cell and promotion of RCT, *via* upregulation of the efflux protein ABCA-1 expression on plaque macrophages and the subsequent cholesterol efflux toward exogenous acceptors (i.e., Apo-E-containing HDL) (Chinetti-Gbaguidi et al., 2011); a decrease in the expression of retention factors (Brodsky and Fisher, 2008); a reduced monocyte recruitment *via* their transformation in monocyte-derived dendritic cells and subsequent upregulation of CC-chemokine receptor (CC-R)-7 on their surface, which allow emigration to the lymphatic system, restoring permeabilization and reducing lymphatic vessel fibrosis (Ivanov et al., 2016). Finally, the retention/migration factors contributing to macrophage loss from the plaque, through reverse transmigration to the lumen or through trafficking to the adventitial lymphatic (Potteaux et al., 2011).

In the context of diabetes, as depicted in Figure 1 (central panel), regression of atherosclerosis is impaired. High glucose levels modulate LXR-dependent gene expression, by inhibiting the LXR-dependent expression of ABCA1, but not ABCG1 (Hussein et al., 2015) and by inducing miR-33, a key negative regulator of the RCT factors, ABCA1 and HDL (Wijesekara et al., 2012). In mouse models of insulin-deficient diabetes, it has been shown that leukocytosis (monocytosis and neutrophilia) is hyperglycemia-dependent. The myelopoiesis is driven by increased expression of certain DAMPs, specifically, signaling through the pattern recognition AGE/RAGE. The relevance to human health and disease is suggested by the correlation between serum S100A8/S100A9 (the associated DAMPs) and the incidence of CAD in a subset of T1D patients from the Pittsburgh EDC study, highlighting the potential importance of glucose control and lipid-lowering therapy as strategies to promote regression of atherosclerosis in diabetics and also suggesting a number of therapeutic targets, including disruption of the S100A8/S100A9-RAGE signaling axis (Nagareddy et al., 2013).

## Diabetic Kidney Disease

### Definition of DKD

DKD typically develops over many decades. It is characterized by progressive proteinuria (microalbuminuria 30–299 mg/24 h to macroalbuminuria > 300 mg/24 h) with a subsequent decline in glomerular filtration reflected by increased serum creatinine (National Kidney Foundation, 2002). The pathophysiology of DKD typically reflects the convergence of hemodynamic, metabolic and inflammatory insults in susceptible individuals (Harjutsalo and Groop, 2014). Current interventions focus on tight glycemic control and RAAS blockade by ACE inhibition or angiotensin receptor antagonism to dilate the efferent arteriole and reduce glomerular hypertension. At best, these interventions slow the progress of disease (Forbes and Cooper, 2013). There is a growing appreciation that oxidative stress and inflammation are key drivers of DKD and may be appropriate targets for therapeutic intervention. Circulating inflammatory cytokine levels correlate with albuminuria and elevated levels of soluble TNF-receptor-1 is an independent predictor of decline in renal function (Krolewski et al., 2014).

### Cellular Pathogenetic Mechanisms of DKD

**FIGURE 2:**
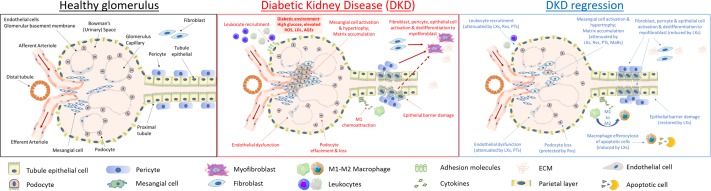
Cellular targets of SPMs in diabetic kidney disease. DKD is typified by mesangial expansion, increased ECM deposition, podocyte loss, renal tubule epithelial cell injury and PMN leukocyte recruitment. Key steps in renal inflammation and fibrosis which may be attenuated by SPMs are highlighted above and include leukocyte recruitment and activation (Godson and Brady, 2000; Leonard et al., 2002; Duffield et al., 2006; Brennan et al., 2018b); renal tubule epithelial cytokine production and dedifferentiation (Kieran et al., 2003; Duffield et al., 2006; Brennan et al., 2013, 2018b); endothelial activation (Baker et al., 2009; Sun et al., 2013); fibroblast to myofibroblast activation (Borgeson et al., 2011; Qu et al., 2012); podocyte effacement (Zhang et al., 2013); macrophage *efferocytosis* and M1:M2 ratio (Mitchell et al., 2002; Borgeson et al., 2011, 2015); mesangial cell activation and matrix accumulation (McMahon et al., 2002; Rodgers et al., 2005; Wu et al., 2006, 2007, 2009; Tang et al., 2014).

The physiological functionality of a healthy glomerulus is outlined in Figure 2 (left). Changes in renal hemodynamics, reflecting glomerular and systemic hypertension, arise early in DKD and lead to glomerular hyperfiltration. RAAS activation leads to increased angiotensin II and endothelin-1 causing efferent arteriolar vasoconstriction and hyperfiltration. Glomerular damage is characterized by podocyte effacement resulting in proteinuria. Renal hypertrophy is also observed in DKD reflecting accumulation of mesangial matrix, glomerular BM thickening and tubular hypertrophy. As matrix expands, it accumulates to form Kimmelstiel–Wilson nodules, a pathological feature of DKD. TIF is considered the major determinant of progression of DKD (Duffield, 2014). The mechanisms underlying TIF have been exhaustively investigated in the context of chronic kidney disease, including DKD (Leaf and Duffield, 2017). At a cellular level, several mechanisms have been proposed including activation of resident fibroblasts to matrix producing myofibroblasts, detachment of pericytes and matrix production, recruitment of fibrocytes from bone marrow and EMT (Kalluri and Weinberg, 2009). Whereas the role of EMT in TIF has been questioned the loss of several epithelial cell markers (de-differentiation) has been observed together with expression of pro-fibrotic mediators such as CTGF and the TGFβ1 activator THBS-1 (Thiery et al., 2009). Experimental evidence suggests that partial EMT and chronic inflammation converge to create a profibrotic *milieu* facilitating collagen production by fibroblasts and recruited hematopoietic cells in the kidney (Zeisberg and Duffield, 2010; Buchtler et al., 2018). Glomerulosclerosis and TIF lead eventually to organ failure and a requirement for renal replacement therapy (hemodialysis or transplantation). Efforts to directly target inflammation in DKD have included manipulating chemokine and cytokine signals in T2D, such as antagonism of CCR2/CCR5 (Huh et al., 2018).

### Molecular Pathogenetic Mechanisms of DKD

As depicted in Figure 2 (central panel), high glucose exerts specific toxic effects on the resident cells of the kidney, including specialized parietal epithelial cells (podocytes), mesangial cells, endothelia, fibroblasts and epithelia driving cellular de-differentiation (Eddy and Neilson, 2006; Liu, 2011). Many of these responses are driven by autocrine and paracrine mediators released by target cells and infiltrating monocytes/macrophages, as typified by responses to TGF-β1 and its downstream targets, including CTGF (Murphy et al., 1999; Strutz et al., 2000; Boor and Floege, 2011). Hyperglycemia leads to ROS production and activation of inflammatory responses including NF-kB and janus kinases and signal transducer and activator of transcription proteins (JAK-STAT) activation and subsequent downstream cytokine production (Sifuentes-Franco et al., 2018).

### Current Therapies in DKD

Numerous large scale genome-wide association studies have been carried out in DKD over recent years (Sandholm et al., 2014; Ahlqvist et al., 2015; Teumer et al., 2015; Wuttke and Köttgen, 2016; van Zuydam et al., 2018). These studies have frequently implicated inflammatory pathways in the pathogenesis of DKD. Such genetic validation of therapeutic targets includes the JAK-STAT pathway. STAT-1,3 activation is observed in renal biopsies from people with DKD (Berthier et al., 2008). Baricitinib, a small molecule JAK-STAT inhibitor, has shown efficacy in a small scale clinical trial. Treatment with baricitinib was associated with decreased inflammatory biomarkers (e.g., urinary chemokine CCL-2, plasma soluble tumor necrosis factor receptor-1, intracellular adhesion molecule-1 and serum amyloid A). Baricitinib decreased albuminuria in participants with T2D and DKD (Tuttle et al., 2018).

Glucose stimulates inflammasome assembly, caspase-1 activation and IL-1β release (Schroder et al., 2010). IL-1β activation by the NACHT, LRR and PYD domains-containing protein (NLRP) inflammasome is an important component of CKD (Vilaysane et al., 2010). Blockade of IL-1β activity in post MI patients with CKD reduced the risk of adverse cardiovascular events among those with CKD (Ridker et al., 2017). As described above, the CANTOS trial targeted IL-1β in atherosclerosis patients and the intervention reduced inflammation and cardiovascular events (Ridker et al., 2011). Comparable effects were observed among those with baseline albuminuria or diabetes. Canakinumab, however, was without effect on serial measures of eGFR, creatinine, the urinary albumin:creatinine ratio or reported adverse renal events during trial follow-up (Ridker et al., 2018).

Despite the identification of numerous drivers of fibrosis, such as the TGF superfamily, thus far efforts to target RF *per se* have been unsuccessful. A recent double blind phase II study assessed whether modulating TGF-β1 activity with a TGF-β1-specific, humanized, neutralizing monoclonal antibody was effective in slowing renal function loss in patients with diabetic nephropathy on RAAS inhibition treatment over a 12-month period. No significant impact on disease progression was observed (Voelker et al., 2017). Other approaches have focused on the balance between BMP family agonist-antagonist activities and promoted BMP-7 and or small peptide mimetics, such as THR-123 (Ali and Brazil, 2014; Tampe and Zeisberg, 2014; Brazil et al., 2015). However, some of these data have been controversial (Sugimoto et al., 2012). Systemic administration of BMP-7 protein is problematic due to the low availability in the kidney, explaining the need for a huge amount of BMP-7 for its reno-protective action, which might exert adverse effects elsewhere (Vukicevic et al., 1998; Yanagita, 2012). CTGF/CCN2 has also been proposed as a potential target (Falke et al., 2014, 2017). Other therapeutic approaches which have been proposed in the context of DKD include attenuation of NF-kB signaling (Lee J. et al., 2012), breakdown of AGEs (Rabbani and Thornalley, 2018) or RAGE antagonism (Bongarzone et al., 2017). In this context, bardoxolone methyl is a novel synthetic triterpenoid belonging to the antioxidant inflammation modulator class. Antioxidant inflammation modulators potently induce the antioxidant and cytoprotective transcription factor Nrf2, reduce the pro-inflammatory activity of the IKK-β/NF-κB pathway, increase the production of antioxidant and reductive molecules, and decrease oxidative stress, thereby restoring redox homeostasis in areas of inflammation. Activation of anti-oxidant responses *via* Nrf2 and inhibition of NF-kB by the triterpenoid bardoxolone methyl reduces oxidative stress, inflammation and promotes mitochondrial function in numerous experimental models of CKD, including DKD (Pergola et al., 2011). Unfortunately, clinical trials of bardoxolone methyl in patients with stage 4 CKD and T2D were prematurely terminated for safety concerns (Tayek and Kalantar-Zadeh, 2013). Bardoxolone methyl treatment was associated with approximately double the risk of heart failure as placebo. Subsequent analysis suggests that these data did not represent toxicity *per se* and that further development of this compound may be warranted with more careful patient selection (Chin et al., 2014, 2018).

### Novel Therapeutic Approaches in DKD

It is important to note some recent advances that suggest renoprotection in response to newer therapeutics which regulate blood glucose and reduce cardiovascular risk in T2D. Intriguingly these reno-protective responses may be independent of glucose lowering. Such interventions include the incretin-based therapeutics (GLP-1-RAs, e.g., liraglutide or DPP-4 inhibitors and SGLT-2 inhibitors) enhancing glycemic control with a low risk of hypoglycemia. However, the use of these agents is limited in those with significant renal impairment. A recent trial treatment with liraglutide, a GLP-1 analog, was associated with a 22% lower incidence of doubling serum creatinine, persistent macroalbuminuria, development of ESRD or death from renal disease relative to controls (Mann et al., 2017). Similar data have been reported for other GLP-1 receptor agonists and for DPP-4 inhibitors which inhibit breakdown of endogenous GLP-1. SGLT-2 inhibitors suppress glucose reabsorption by the proximal tubule and therefore increase glucose excretion. The SGLT-2 inhibitors target reabsorption of both glucose and sodium as a result there is increased sodium delivery to the *macula densa* activating tubule-glomerular feedback afferent arteriolar vaso-modulation, resulting in increased renal blood flow and decreased glomerular hyperfiltration. SGLT-2 inhibition is associated with lower rates of albuminuria and lowering rates of eGFR decline (Tomkin, 2014). Intriguingly, bariatric surgery in T2D appears to have specific reno-protective effects which may relate to enhanced GLP-1 responsiveness (Docherty and le Roux, 2014). Miras et al. (2015) reported that, 1-year post-bariatric surgery, a decrease urinary albumin/creatinine ratio was observed whereas no benefit was seen on other microvascular complications, i.e., retinopathy or neuropathy. SGLT-2 inhibitors and GLP-1 targeting drugs attenuate inflammatory responses in DKD. As we will discuss below, we propose that exploiting the bioactivity of endogenous lipid modulators that promote the resolution of inflammation and suppress fibrosis is a novel therapeutic paradigm worthy of consideration as adjuvant therapy in DKD.

## The Role of Macrophage in Diabetes Complications

As described, macrophages are key players in atherosclerotic lesions, regulating the local inflammatory milieu and plaque stability by the secretion of many inflammatory molecules, growth factors and cytokines (Wolfs et al., 2011). The macrophage paradigm classically reflects the heterogeneity of their monocyte progenitor: alternative crawling monocytes continuously patrol the endothelium of blood vessels in the steady state. The patrolling monocytes rarely extravasate in the steady state. In contrast, during inflammation or infection, classical monocytes are the first to extravasate when inflammation signaling occurs, and, within few hours they differentiate in M1 “pro-inflammatory macrophages,” induced by INFγ + LPS or by TNFa, characterized by a high phagocytic profile. At later stages of inflammation, non-classical monocytes (or *non-phlogistic* monocytes) *trans*-migrate and initiate a differentiation program into ‘M2’-like macrophages, which play a role in resolving of inflammation and tissue repair (Geissmann et al., 2008). M2 macrophages can polarize toward different phenotypes according to various stimuli present in their surrounding micro-environment (Mosser and Edwards, 2008) and to their distinct gene expression profiles (Mantovani et al., 2004). In particular, “*M2a*” or “*alternative*” macrophage is the product of Th2 activation (by IL4 and IL13 cytokines or fungal and helminth infections) and is responsible for a type II inflammatory response (consisting in killing parasites and inducing a Th2 response to allergy). “*M2b*” or “*type II*” macrophage is elicited by IL-1 receptor ligands, immune complexes and LPS, triggering the activation of Th2 system. “*M2c*” or “*deactivated*” macrophage is induced by IL10, TGF-β and glucocorticoids and is mainly immunoregulatory, through matrix deposition and tissue re-modeling (Martinez and Gordon, 2014). A fourth type, “*M2d*,” or “*angiogenic*” macrophage is elicited by IL-6 and adenosine and is mainly involved in wound healing (Ferrante and Leibovich, 2012). However, recent findings provide evidence for proliferation of local macrophages or *trans*-differentiation from other vascular cells as alternative sources (Nagenborg et al., 2017). In particular, it has been shown that cholesterol-loading induces the *trans*-differentiation of SMCs to macrophage-like cells (Rong et al., 2001) and more recently, it has been shown that approximately 50% of foam cells might have a SMC origin (Allahverdian et al., 2014). Linear tracing studies from Randolph’s lab have shown that tissue-specific factors drive highly specialized macrophage functions irrespective of their ontological origin, suggesting tremendous plasticity and redundancy in the mononuclear phagocyte system. Whether embryonic and adult macrophages possess specialized roles has yet to be formally tested. However, the conceptual understanding and genetic tools are now sufficiently developed to precisely follow both embryonic and adult macrophage subsets in health and disease, which should allow important and unanswered questions in the field to be addressed. In order to develop novel therapies, a critical future goal is to harness this new found understanding that different macrophage lineages exist within tissues and clarify whether these distinct lineages differentially contribute to tissue damage and repair (Epelman et al., 2014).

### Role of Macrophage in DAA

In the context of atherosclerosis, macrophages uniquely possess a dual functionality, regulating lipid accumulation and metabolism and sustaining the chronic inflammatory response, two well-documented pathways associated with the pathogenesis of the disease (Moore and Tabas, 2011).

Established atherosclerotic plaques from patients with existing CAD undergoing carotid endarterectomy classified as *symptomatic* (where the patient has experienced previous ischemic events but without any CVD diagnosis) or a*symptomatic* (where a patient has no history of ischemic events or CVD) have been recently comprehensive histologically and immunohistochemically characterized for their cellular content and macrophage subsets of atherosclerotic lesion. Symptomatic plaques were defined as highly hemorrhagically active and the internal carotid was the most diseased segment, based on the predominant prevalence of fibrotic and necrotic tissue, calcifications, and hemorrhagic events. Immunohistochemical analysis showed that both M1 and M2 macrophages are present in human plaques. However, M2 macrophages were localized to more stable locations within the lesion. Importantly, M1 markers and Th 1-associated cytokines were highly expressed in symptomatic plaques, whereas expression of the M2 markers, mannose receptor and CD163 and Th2 cytokines were inversely related with disease progression (de Gaetano et al., 2016). A strong relation between macrophage, mitochondria and glucose dysregulation has recently emerged in a number of studies from Fredman and Tabas (2017). Clearance of ACs by phagocytes (*efferocytosis*) prevents post-apoptotic necrosis and dampens inflammation. Mitochondrial fission in response to AC uptake is a critical process that enables macrophages to clear multiple ACs and to avoid the pathologic consequences of defective efferocytosis *in vivo* (Yurdagul et al., 2017).

### Role of Macrophage in DKD

In a renal context, macrophages constitute a major subset of the infiltrating inflammatory cells and their contribution to renal fibrogenesis is well established (Duffield, 2010). Macrophage infiltration has been found to correlate with TIF on kidney biopsies (Young et al., 1995), and to correlate negatively with outcome in CKD of diverse etiologies (Tinckam et al., 2005; Duffield, 2010). However, the role of macrophages in this context is not entirely clear-cut. M1 macrophages are recruited to the kidney at early time points in a murine IRI model, whereas at later time points, M2 macrophages predominate. Additionally, in this model, depletion of macrophages prior to IRI has been found to attenuate inflammation and TIF, whereas macrophage depletion after 3–5 days is shown to slow tubular cell proliferation and repair (Lee et al., 2011). Macrophages exhibit some plasticity, and may not remain committed to a single phenotype. As a component of the programmed resolution of inflammation, a phenotypic change is triggered by altered cytokine and lipid mediator profiles in the microenvironment, and M1 phenotype macrophages thus ‘switch’ to a pro-resolving M2 phenotype (Nathan and Ding, 2010; Lee et al., 2011). In the context of chronic inflammation, or repeated injuries, the factors that determine if macrophages are predominantly reparative *versus* predominantly pro-inflammatory remain unclear. Directing more of the macrophage population toward a pro-resolving phenotype may provide a novel therapeutic approach in CKD. Although much of our current understanding of the ontogeny and functional plasticity of macrophages has been derived from murine models, it is important to note that, together with the above mentioned contribution from Randolph on macrophage ontology, a recent study in human heart reveals two populations of macrophages with different origins and functions: CCR2 expressing macrophages are recruited from bone marrow and proliferate and are functionally proinflammatory and abundant in regions of scarring, whereas macrophages lacking CCR2 are maintained by local proliferation and express genes associated with tissue repair (Bajpai et al., 2018).

## Diabetic Retinopathy: A Brief Overview

### Definition, Pathogenesis, and Current Therapies

Diabetic retinopathy (DR) is a microvascular complication of diabetes, clinically characterized by progressive alterations in the microvasculature that lead to retinal ischemia, neovascularization, altered retinal permeability and macular edema. It is currently the leading cause of blindness in the adult working population (Congdon et al., 2004; Yau et al., 2012).

The disease can be divided into two main stages – non-proliferative retinopathy and proliferative retinopathy distinguished by the absence or presence of abnormal neovascularization, respectively. The final stage “proliferative retinopathy” is characterized by neovascularization of the disk or iris or vitreous hemorrhage or retinal detachment (Wilkinson et al., 2003). Macular edema or “diabetic maculopathy” can occur at both the non-proliferative and proliferative stages as a result of fluid accumulation under the macula.

The pathological and morphological alterations associated with DR were long considered to be primarily microvascular in nature, as a result of hyperglycemia and the metabolic pathways it activates. The onset of clinically detectable DR is characterized by changes in the micro-vessels of the eye which includes thickening of the BM, loss of vascular permeability, loss of pericytes, capillary occlusions and microaneurysms (Xu et al., 2014).

However, recent studies have demonstrated that retinal neurodegeneration is a critical feature associated with the progression of the disease and may in fact precede the development of clinically detectable microvascular damage (Lieth et al., 2000; Puro, 2002).

Under pathological DR conditions, break-down of the BRB occurs as a result of the presence of increased levels of vascular permeability factors, such as VEGF in the vitreous of the eye. The resulting “leaky” vasculature leads to increased albumin flux into the retina and fluid accumulation resulting in macular edema and possible vessel hemorrhage (Klaassen et al., 2013).

Thickening of the vascular BM occurs early in the disease and represents one of the first histologically detectable structural alterations. Several biochemical alterations contribute to BM thickening *in vivo*. Increased expression of the matrix components of the BM, including fibronectin (Roy et al., 1996), collagen IV (Roy et al., 1994) and laminin (Ljubimov et al., 1996) can be detected long before the formation of diabetic lesions. BM turnover is tightly regulated by the delicate balance of synthesis and degradation of BM components by MMPs, urokinases and their inhibitors. This balance is disturbed during DR (Kowluru et al., 2012).

Several interconnected biochemical pathways associated with hyperglycemia have been implicated in the pathogenesis of DR, including increased polyol pathway flux, increased hexosamine pathway flux and activation of protein kinase C. A crucial role is played by hyperglycemia-induced ROS production and AGEs formation (Forbes and Cooper, 2013). The retina is the most metabolically active tissue in the body, rendering it particularly susceptible to oxidative stress (Wu et al., 2014). Although all retinal cells express RAGE ubiquitously, retinal pericytes, in particular, have been shown to accumulate AGEs, contributing to BRB breakdown, which is in part accredited to pericyte loss, but also to AGE-induced leukocyte adherence to retinal ECs (Moore et al., 2003).

Growing consensus is emerging in the predominant role of inflammation in the pathogenesis of DR (Rubsam et al., 2018). The formation of AGEs and the activation of PKC have been implicated in the activation of pro-inflammatory mediators, such as NF-κB, connecting hyperglycemic-induced oxidative stress to inflammation. An increase in a number of pro-inflammatory cytokines and chemokines has been demonstrated in both diabetic patients and models of experimental retinopathy (Doganay et al., 2002; Sato et al., 2009). Blocking the activity of pro-inflammatory cytokines (such as TNF-α, IL-6, and IL-1) has shown beneficial effects in models of retinopathy. An IL-1 receptor antagonist reduces inflammatory responses in a rodent model of T2D (Vallejo et al., 2014) while breakdown of the BRB was completely ablated in a TNF-α knockout diabetic mouse (Huang et al., 2011). Chemokines, such as MCP-1 and IL-8, are also elevated in diabetic eye disease and contributed to neovascularization and fibrosis (Yoshida et al., 2003). However, their expression was reduced by inhibitors of VEGF, suggesting that the action of both MCP-1 and IL-8 are mediated through pathways involving VEGF. Hyperglycemic conditions also drive increased expression of a number of growth factors (including VEGF and TGFβ) mediating the retinal damage associated with DR, such as BM thickening, vascular permeability and neovascularization.

TNF-a and VEGF have received particular attention for their role in the vascular lesion and neovascularization associated with late stage retinopathy. Therefore, anti-TNFa (i.e., Infliximab) (Sfikakis et al., 2005) and anti-VEGF (i.e., Avastin) (Haritoglou et al., 2006) intravitreal therapies are standard clinical therapeutic options for the treatment of DR.

Many of the agents developed to target the various biochemical pathways driven by hyperglycemia have had limited effect clinically, pointing to a need for new therapeutics targets.

## The Role of SPMs in Resolving Inflammation

The inflammatory response consists of two phases: initiation and resolution. The initiation phase is characterized by the site-specific accumulation and coordinated activation of a host of immune effector cells in an inflammatory cytokine and pro-inflammatory lipid mediator rich environment. Inflammation is critical in the host response to infection and injury, however, timely resolution is necessary for the restoration of tissue homeostasis, thereby limiting excessive tissue injury, preventing the development of a chronic inflammatory state (Serhan et al., 2007). Non-resolving inflammation is a major driver of disease. Multiple mechanisms ensure physiological resolution toward tissue homeostasis. Cells like macrophages switch phenotypes by secreting molecules like reactive oxygen intermediates, lipids and proteins which impact a cell from displaying pro- or an anti-inflammatory behaviors (Buckley et al., 2014).

Whereas inflammation and its effective outcome, i.e., a return to homeostasis, were typically considered a manifestation of the passive dissipation of pro-inflammatory stimuli, including lipids, such as prostaglandins and leukotrienes, it is now clear that the resolution of inflammation is an active and dynamically regulated process reflecting responses to endogenously generated mediators, including cytokine and lipids (Godson and Brady, 2000; Maderna and Godson, 2009; Serhan, 2014).

The specialized SPMs are a family of endogenously produced pro-resolving lipid mediators derived from the metabolism of PUFAs, which include LXs, resolvins (Rvs), protectins (PDs) and MaRs. They were discovered by Serhan et al. (1984). LXs (*Lipoxygenase interaction products*) were firstly isolated in a human leukocyte (Serhan et al., 1984) and classified as derivatives of the ω6 fatty acid arachidonic acid (20:4, n-6). Rvs (*Resolution phase interaction products*) were firstly identified in a resolving inflammatory exudate in 2000 (Serhan et al., 2000), PDs (termed neuroprotectin D1 if generated in neural tissue for its protection in neurons, glial cells, and brain stroke; or protectin D1 for other tissue in 2004 (Bazan, 2005) and MaRs (*Macrophage mediator in Resolving Inflammation*) in 2009 (Serhan et al., 2009). Rvs, PDs and MaRs are classified as derivatives of ω3 fatty acids: specifically, Rvs can either form from the EPA (20:5, n-3) [RVs E-series] or from the DHA (22:6, n-3) [RVs D-series]; while, PDs and MaRs only derive from DHA. As their precursors, all these derivatives are classified as PUFAs and they demonstrated potent anti-inflammatory and immunoregulatory actions (Serhan et al., 2008).

Within a few hours from barrier break, tissue injury or trauma, eicosanoids are crucial in initiating the cardinal signs of inflammation (redness, heat, pain and swelling). As part of the vascular response, leukocytes traffic to the site of injury. The prostaglandins PGE_2_ and PGI_2_ (involved in vasodilation) and the leukotriene LTB_4_ (involved in chemotaxis and adhesion) stimulate the migration of PMN to the tissue. In parallel to the PMN–monocyte sequence, lipid mediator composition of the inflammatory exudate switches class, from eicosanoids to SPMs, marking the beginning of the end of the acute inflammatory response. LXs are the first SPM to be locally produced, highlighting its role as “stop” signal to eicosanoid production (*exudate switch*), as firstly described by the work of Levy et al. (2001). LXs and Rvs also stimulate the recruitment of monocytes. The resolving macrophages then clear apoptotic PMNs, inflammatory debris by *efferocytosis* (stimulated by LXs, Rvs, PDs). After this has taken place, normal structure and homeostasis can be restored. “Resolution” is defined as the period between peak inflammatory cell influx and the clearance of these cells from the tissue site and the restoration of functional homeostasis. Subsequent post-resolution events involves activation of adaptive immunity B- and T-lymphocytes (Fullerton and Gilroy, 2016).

Failed resolution can lead to increased levels of prostaglandins and leukotrienes, chronic inflammation and fibrosis. Ultimately SPMs reduce the magnitude and duration of inflammation (Arita et al., 2007), stimulate re-epithelialization (Hellmann et al., 2018), wound healing (Dalli, 2017) and tissue regeneration (Dalli, 2017).

While most of the studies involving SPMs have been conducted on rodents models, major and recent advances have been represented by the work of Motwani et al. (2016) in humans, where a new translational model of self-resolving acute inflammatory response triggered by the intradermal injection of UV-killed Escherichia coli into the forearm of healthy volunteers was described. For the first time SPMs endogenous production have been identified in humans over the course of the inflammatory response. It has also been shown that resolution is an active process accelerated by addition of exogenous SPMs.

The molecular mechanisms through which SPMs exert their responses include activation of distinct GPCRs and regulation of gene expression. The binding, and consequent activation, of the LX/N-formyl peptide receptor-2 (ALX/FPR2) GPCR by lipids, such as LXA_4_ and RvD1 as well as Annexin-1 peptide (Krishnamoorthy et al., 2010; Maderna et al., 2010; Bena et al., 2012), and the RvE1 agonism at the ChemR23 GPCR (Arita et al., 2007) are key to reduce PMN infiltration and subsequently stimulate *efferocytosis* by macrophages, heralding the initiation of pro-resolving cascade of events.

## SPMs in Acute Injuries

The anti-inflammatory and pro-resolving properties of SPMs, including LXs, Rvs and their mimetics, particularly 15(R/S)-methyl-LXA_4_ (Wu et al., 2013), benzo-LXA_4_ (Sun et al., 2009), BDA-RvD1 (Orr et al., 2015) and have been demonstrated in several types of experimental acute renal and peritoneal injury (see below).

Moreover, SPMs have recently been shown to play a key role in dampening both sterile inflammation and infection (or non-sterile inflammation). In this context, a recent advance is represented by the above mentioned study on the self-resolving properties of SPMs in an acute and local *E. coli*-induced translational skin-blisters model (Motwani et al., 2016).

### Biosynthesis and Functions of LXs in Acute Injuries

Native LXs, LXA_4_ and LXB_4_, are endogenous eicosanoids, transcellularly biosynthesized by 5- and 15-LO interaction of activated leukocytes with epithelium, endothelium or platelets (Serhan et al., 1984; Serhan, 1989; Serhan and Sheppard, 1990) Acetylation of cyclooxygenase-2 by aspirin can trigger the biosynthesis of their 15R-carbon epimers, 15-epi-LXA_4_ and 15-epi-LXB_4_ [15-epi-LXs or aspirin-triggered LXs (ATLs)] (Serhan, 2005). Although native LXs have demonstrated potent anti-inflammatory and pro-resolution bioactions (Claria et al., 1996; Fierro and Serhan, 2001; Chandrasekharan and Sharma-Walia, 2015), their therapeutic potential is compromised for by their chemical instability and for by their rapid metabolic inactivation by prostaglandin dehydrogenase-mediated metabolic inactivation *in vivo* (Clish et al., 2000), with the growing need to synthesize their mimetics.

First-generation synthetic LXA_4_ analogs were designed in 1995–1998 by Serhan, Petasis and colleagues to minimize metabolism of the molecule (Parkinson, 2006). These relatively stable pharmacological agents, together with myeloid-specific ALX-R-expressing transgenic mice, have provided powerful tools to explore LX functions *in vivo*. Among those, pharmacokinetic analysis of *ATLa*, such as methyl (5R,6R,7E,9E,11Z,13E,15S)-16-(4-fluorophenoxy)-5,6,15-trihydroxy-7,9,11,13-hexadecatetraenoate, revealed β-*oxidation* as a novel route for LXA_4_ metabolism, prompting the development of second-generation 3-oxa-LXA_4_ analogs with improved pharmacokinetic disposition (Parkinson, 2006).

Second-generation *3-oxa-LXA_4_ analogs*, such as (5R,6R,7E,9E,11Z,13E,15S)-16-(4-fluorophenoxy)-3-oxa-5,6,15-trihydroxy-7,9,11,13-hexadecatetraenoic acid, have shown potency and efficacy comparable to ATLa in diverse animal models after topical, intravenous or oral delivery (Guilford and Parkinson, 2005).

More recently, a new class of LX-analogs featuring a benzo-fused ring system have been designed and proved to be as potent as native LXA_4_ in a series of *in vitro* and *in vivo* studies (O’Sullivan et al., 2007; Petasis et al., 2008). In particular, it was found to stimulate phagocytosis of apoptotic PMN by macrophages, in a zymosan-induced peritonitis murine model of acute inflammation (O’Sullivan et al., 2007). Further exploration of the mechanism of action through which PMN phagocytosis by bone marrow-derived macrophage was elicited revealed that expression, activation and internalization of ALX/FPR2 by LXA_4_ and the glucocorticoid-derived Annexin A1 peptide (Ac2-26) were essential (Maderna et al., 2010).

In early 2000, the work from Leonard et al. (2002) suggested a framework for understanding SPMs bioactions in renal IRI and the molecular basis for renoprotection by LXs in this setting. They firstly demonstrated, in a murine renal IRI, that the stable synthetic LXA_4_ analog 15-epi-16-(FPhO)-LXA_4_-Me is reno-protective, as gauged by lower serum creatinine, attenuated leukocyte infiltration and reduced morphologic tubule injury. Subsequently, they employed complementary oligonucleotide microarray and bioinformatic analyses to probe the transcriptomic events that underpin LX renoprotection and found that epi-LXA_4_ modified the expression of many differentially expressed pathogenic mediators, including cytokines, growth factors, adhesion molecules and proteases. Importantly, this LX-modulated transcriptomic response included many genes expressed by renal parenchymal cells (such as the Claudin family epithelial tight junctions) (Kieran et al., 2003).

### Biosynthesis and Functions of Rvs in Acute Injuries

Rvs are produced by 12/15-LO, p450, and/or 5-LO, in *trans*-cellular or intracellular biosynthetic systems of leukocytes or leukocytes plus endothelia/epithelia (Serhan et al., 2000). The novel lipid mediators produced from EPA were first isolated from resolving exudates that proved to contain 18R-HEPE as well as several other related bioactive compounds and were therefore collectively named 18R-E series (Serhan et al., 2000). The first bioactive product isolated from exudates, coined RvE1, reduced inflammation and blocked human PMN *trans*-endothelial migration.

RvDs are derived from DHA. During inflammation, endogenous DHA is converted to 17S-HEPE which are then converted in 17S-hydroxyl-containing RvDs (RvD1–RvD6) and docosa-conjugated triene-containing PD1/NPD1, *via* 15-LO (15S-lipoxygenation)-initiated biochemical pathways (Serhan et al., 2002; Hong et al., 2003; Marcheselli et al., 2003) or to 14S hydroxyl-containing MaRs *via* 12-LO (12S-lipoxygenation)-initiated biochemical pathways. 5-LO catalyzes sequentially with 15-LO or 12/15-LO, generating RvDs (Hong et al., 2003) and some MaRs (Serhan et al., 2009).

RvD1 is converted by eicosanoid oxidoreductases to 17-oxo-RvD1 and 8-oxo-RvD1. The former is an inactive metabolite, while the latter is still effective in suppressing PMN infiltration (Sun et al., 2007). RvE1 is metabolized to 12-oxo-RvE, 18-oxo-RvE1, 10,11-dihydroxy RvE, 19-hydroxy RvE1, 20-hydroxy RvE1 in tissue or cells, of which the first four metabolites are inactive partially or completely in inflammation resolution, and thus are representative of RvE1 metabolic deactivation (Arita et al., 2006; Hong et al., 2008). Human PMNs convert PD1 to its omega-22 hydroxy product (Serhan and Petasis, 2011). The metabolic deactivation of Rvs dysregulated in pathological conditions, may result in their deficiency, or in diminishing the pharmacological efficacy of administered resolvins. Therefore, a series of stable analogs have been successfully synthesized, such as a *p*-fluorophenoxyl added to RvE1 and RvD1 ω-terminal, which blocks the critical metabolic inactivation of RvE1 or RvD1 without attenuating the anti-inflammatory pro-resolving activities (Arita et al., 2006; Hong et al., 2008; Tang et al., 2014) In particular, the RvE1 analog 19-(p-fluorophenoxy)-RvE1 was synthesized to resist rapid metabolic inactivation and proved to retain biological activity reducing PMN infiltration and pro-inflammatory cytokine/chemokine production *in vivo*. These results established the structure of a novel RvE1 initial metabolite, indicating that conversion of RvE1 to the oxo product represents a mode of RvE1 inactivation. Moreover, the designed RvE1 analog, which resisted further metabolism/inactivation, could be a useful tool to evaluate the actions of RvE1 in complex disease models (Arita et al., 2006).

These lipids act as paracrine and autocrine mediators of leukocytes to promote resolution of acute injuries, including AKI-initiated inflammation and fibrosis and rescue of kidney functions (Zhao et al., 2016), by shortening PMN life span and promoting macrophage *efferocytosis* of ACs and the subsequent exit of the phagocytes from inflammatory tissue.

RvD1 and RvE1 also switch macrophage to the phenotype that produces pro-resolving interleukin-10. RvDs or protectin/neuroprotectin D1 (PD1/NPD1) inhibits PMN infiltration into injured kidney, blocks TLR-mediated inflammatory activation of macrophage and mitigates renal dysfunction. RvDs also repress renal interstitial fibrosis, and PD1 promotes reno-protective heme-oxygenase-1 expression. These findings provide novel approaches for targeting inflammation resolution and LMs or modulation of LM-associated pathways for developing better clinical treatments for AKI. Moreover, in LPS-induced AKI, RvD1 could decrease TNFα level, ameliorate kidney pathological injury, protect kidney function, and improve animal survival by down-regulating NFκB inflammatory signal as well as inhibiting renal cell apoptosis (Zhao et al., 2016). Intriguingly, RvE1 counter-regulates leukocytes partially *via* increased LXA_4_ biosynthesis (Levy et al., 2011). Since AKI is the major complication of renal allograft transplantation (Bellomo et al., 2012), these results further demonstrate the effectiveness of LXA_4_ or RvE1 in reducing AKI. LX actions converge with the pro-resolving characteristics of RvD1, as LXA_4_ and RvD1 both activate the same GPCRs ALXR/FPR2 and GPR32.

## SPMs in Chronic Diabetes Complications

Unresolved inflammation drives the development of clinically relevant chronic diseases. Here, we focus the attention on the role of SPMs, particularly LXs and Rvs, on DAA, CKD and, briefly, on DR.

As discussed previously, sustained, non-resolved low-grade inflammation, over decades, promotes formation of atherosclerotic lesions characterized by large necrotic cores, thin fibrous caps and thrombosis. In advanced atherosclerosis, there is an imbalance between levels of SPMs and proinflammatory lipid mediators, which results in sustained leukocyte influx into lesions, inflammatory macrophage polarization, and impaired *efferocytosis*. In animal models of advanced atherosclerosis, restoration of SPMs limits plaque progression by suppressing inflammation, enhancing *efferocytosis*, and promoting an increase in collagen cap thickness (Fredman and Tabas, 2017).

From a CKD-perspective, there is a clear mechanistic link between non-resolving inflammation and fibrosis. Non-resolving inflammation results in sustained secretion of pro-fibrotic cytokines and other inflammatory mediators from both resident and infiltrating cells, eliciting fibroblast proliferation and epithelial cell de-differentiation. Sustained or unresolved inflammation is recognized to be an underlying component of many chronic disease states in diverse organ systems, including CKD (Serhan, 2014; Brennan E.P. et al., 2017).

### The Role of LXs and Rvs in Atherosclerosis and DAA

It is now established that the local LO-induced biosynthesis of lipid mediators, including LXA_4_, RvD1 and PD1, protects against atherosclerosis. These mediators exert potent agonist actions on macrophages and vascular ECs that can control the magnitude of the local inflammatory response (Merched et al., 2008), as depicted in Figure 1 (left).

Enhanced biosynthesis of LXA_4_ in transgenic mice is associated with decreased lesion formation in models of atherosclerosis (Merched et al., 2008). Atheroprotective responses of macrophages and ECs to SPMs include enhanced *efferocytosis* of apoptotic debris and modulation of adhesion molecules expression (VCAM-1, ICAM-1, P-Sel). It has been shown that LXA_4_ increases ABCA1 expression and promotes cholesterol efflux through LXRα pathway in THP-1 macrophage-derived foam cells (Sha et al., 2015). Moreover, it has been recently demonstrated that ATL signals through FPR2/ALX in vascular SMCs and protects against intimal hyperplasia after carotid ligation (Petri et al., 2015).

Over the past few years, Brennan’s work focused on the role of miR in both DKD (see details below) and DAA. The let-7 miRNA family plays a key role in modulating inflammatory responses. Vascular SMC proliferation and EC dysfunction are critical in the pathogenesis of atherosclerosis, including in the setting of diabetes. The therapeutic potential of LXA_4_-induced restoration of let-7 mimic levels was observed *in vitro* in SMCs, *in vivo via* tail vein injection in a 24 h murine model, and *ex vivo*, where significant changes to the secretome in response to let-7 therapy were seen. It has been proposed that restoration of let-7 expression, a mimic of response to LXA_4_, could provide a new target for an anti-inflammatory approach in diabetic vascular disease (Brennan E. et al., 2017). Very recently, LXA_4_ and the synthetic LX mimic benzo-LXA_4_ have also been shown to be athero-protective in murine model of DAA (STZ-induced diabetic ApoE^-/-^ mouse). Here there was significant reduction in plaque area. The authors also demonstrated that these SPMs could attenuate vascular SMCs migration and proliferation, EC-monocytes interactions, as well as modulate the pro-inflammatory secretome signature in human carotid plaque explants. Of particular note was the finding that LX treatment reduced pre-existing plaque burden in diabetic mice (Brennan et al., 2018a).

Oxidation of native LDLs plays an important role in the development of atherosclerosis. A very recent work showed that although ox-LDLs are known to be pro-inflammatory and deleterious in the context of atherosclerosis, they are also able to induce a pro-resolution effect by self-induction of RvD1 from HMEC (Dufour et al., 2018). Moreover, circulating inflammation-resolving lipid mediators RvD1 and DHA are decreased in patients with acutely symptomatic carotid disease (Bazan et al., 2017). Similarly, RvE1 and ATL plasma levels were found to be significantly lower in symptomatic peripheral arteries disease than in healthy controls (Ho et al., 2010).

In addition to lipid agonists, the ALX/FPR2 can also bind peptides, such as Annexin-1 (Maderna et al., 2010). In an advanced model of atherosclerosis, the Annexin-1 derivative acetylated peptide (Ac2-26), was delivered using Collagen IV-targeted nanoparticles and it showed therapeutic effect in fat-fed LDL-R^-/-^ mice, including an increase in the protective collagen layer overlying lesions, suppression of oxidative stress and a decrease in plaque necrosis, thus, suggesting a new form of therapy (Fredman et al., 2015).

### The Role of LXs in CKD and DKD

Advances in understanding the effects of LXs in the context of RF arose from investigating their actions on the main cell types involved in kidney failure (mesangial cells, fibroblasts, epithelia, adipocytes) (see details below). As outlined in Figure 2 (right), work from Rodgers, McMahon and Mitchell investigated the potential of LXA_4_ to regulate PDGF-induced gene expression and the associated autocrine TGFβ1 production in human renal mesangial cells, and found that LXA4 is a potent modulator of matrix accumulation and pro-fibrotic change, thus suggesting a potential protective role in progressive renal disease (McMahon et al., 2002; Mitchell et al., 2004; Rodgers et al., 2005). In an experimental model of RF, i.e., unilateral ureteric obstruction (UUO), LXA_4_ and its synthetic benzo-analog attenuated injury by inhibiting TGFβ1-induced fibroblast activation, proliferation and gene expression (Borgeson et al., 2011).

*Aging*, defined as a state of chronic, low-grade, sterile inflammation (*inflamm-aging*) (Franceschi et al., 2017) and *adiposity*, have recently been proposed as one of the major risk factors underlying the pathophysiological development of obesity-associated complications, including T2D, and its complications DAA and DKD (Todd et al., 2015). Therefore, of particular relevance in the diabetes context is the work that Borgeson et al. (2011) subsequently carried out, in 2012 and 2015, on the effect of the native LXA_4_ on obesity-induced adipose tissue inflammation and related diseases. Firstly, using a model of age-associated adipose inflammation, *inflamm-aging* it was shown that LXA_4_ attenuates adipose inflammation, decreasing IL-6 and increasing IL-10 expression. The altered cytokine milieu correlated with increased the insulin-regulated glucose transporter-4 and the insulin receptor substrate-1 expression, suggesting improved insulin sensitivity. Further investigations revealed the ability of LXA_4_ to rescue macrophage-induced desensitization to insulin-stimulated signaling and glucose uptake in cultured adipocytes, thus suggesting that LXA_4_ may represent a potentially useful and novel therapeutic strategy to subvert adipose inflammation and insulin resistance, key components of T2D (Borgeson et al., 2012). Later on, the role of LXs in obesity-related pathologies was further explored by investigating their impact on impaired glucose tolerance, adipose inflammation, fatty liver and CKD. In particular, LXs attenuated obesity-induced CKD, reducing glomerular expansion, mesangial matrix and urinary H_2_O_2_. These data suggested a protective role for LXs against obesity-induced systemic disease, and supported a novel therapeutic paradigm for treating obesity and associated pathologies, such as TD2 and its related complications (Borgeson et al., 2015). A role in the context of aging-related pathologies (including obesity, atherosclerosis, renal disease and diabetes) for SPMs has been also recently reviewed by Doyle et al. (2018).

Certain miRs have been implicated in fibrosis (both renal and cystic). In cultured HK-2 cells, LXA4 suppresses TGF- 1-induced RF through a mechanism involving upregulation of the miR let-7c and downregulation of TGF R1. Expression of let-7c targets is dysregulated in human RF (Brennan et al., 2013). The effects of let-7 on TGF 1-mediated responses of renal epithelia have also been shown by others, including Cooper and Kantharidis, leading to the proposal that let-7b miR represents a potential new target for the treatment of RF in diabetic and non-diabetic nephropathy (Wang et al., 2014; Kantharidis et al., 2015; Brennan E. et al., 2017). Interestingly, LXA4 demonstrated to attenuate TGF- 1-induced fibrotic responses whereby epithelial cells express mesenchymal markers (Brennan et al., 2013). In cultured renal epithelia upregulation of thrombospondin and CTGF is a well-documented fibrotic response (Liu et al., 2013). While, in cystic fibrosis, miR181b is indeed downregulated by LXA4 and RvD1, through ALX/FPR2 activation (Pierdomenico et al., 2015). Moreover, very recent interesting observations showed that LXs can also reverse established atherosclerosis (Brennan et al., 2018a) and DKD (Brennan et al., 2018a).

Very recently, in a DKD murine model, Brennan has also identified a series of transcripts regulated by LXA_4_ and Benzo-LXA_4_, modulating well established (TGF-β1, PDGF, TNF-α, NF-κβ) and novel (early growth response-1) networks in DKD, demonstrating that LXs can reverse established diabetic complications and supporting a therapeutic paradigm to promote the resolution of inflammation (Brennan et al., 2018b). Interestingly, a recent study from Goicoechea measured circulating level of ATL in patients with diabetic and non-diabetic kidney disease and found that diabetes was associated with lower levels of the SPMs and that this could be restored by 12-month low dose aspirin treatment (Goicoechea et al., 2017).

### The Role of Rvs in Diabetic Wound Healing

The work from Spite greatly deepened the knowledge around the SPMs properties of re-epithelialization and/or re-vascularization post ischemia, particularly focussing on Rvs bioactions. RvD2 stimulates arteriogenic revascularization in a murine model of hind limb ischemia suggesting that resolvins may be a novel class of mediators that both resolve inflammation and promote arteriogenesis (Zhang et al., 2016), a mechanism which can provide protection against nephropathy and atherosclerosis.

Altered resolution of acute inflammation in the context of obesity and diabetes, in which PMN apoptosis is delayed and macrophage efferocytosis is defective, cause persistent leukocyte and AC accumulation and defective wound closure (Baltzis et al., 2014). Wound healing in diabetes is enhanced by RvD1 and RvE1 *via* the promotion of macrophage-mediated AC clearance and re-epithelialization (Bannenberg et al., 2005; Spite et al., 2014). Moreover, RvD1 decreases adipose tissue macrophage accumulation and improves insulin sensitivity in obese-diabetic mice, suggesting that RvD1 could provide a novel therapeutic strategy for treating obesity-induced diabetes (Hellmann et al., 2011).

### The Role of LXs and Rvs in DR

Although the anti-inflammatory (anti-TNFα and anti-VEGF) approach is still the standard therapy for DR, recent *in vitro* and *in vivo* models are shifting the attention toward a pro-resolving novel strategy (Das, 2013; Wang and Daggy, 2017).

Since corneal, retinal neuronal degeneration (Srinivasan et al., 2017), conjunctivitis (Stuebiger et al., 2015) and uveitis (Sivaraj et al., 2009) have been associated with DR, the effects of LXs (Gronert, 2005; Biteman et al., 2007; He et al., 2011; Hodges et al., 2017) and Rvs (Tian et al., 2009; Settimio et al., 2012; Li et al., 2013; Lee et al., 2015) in dampening DR are of relevance.

In a well established *in vivo* model of STZ-induced Diabetes, hyperglycemia induces persistent inflammation and tissue damage, due to decreased expression of heme-oxygenase (HO) in the ciliar body (Rossi et al., 2006). Recently, the effect of RvD1on STZ-induced DR has been explored. RvD1 regulates the NLRP3 inflammasome and NFκB signaling pathway (Yin et al., 2017).

Moreover, by using an *in vivo* deletion of 12/15-LOX model, associated with exacerbated inflammation and impaired wound healing, due to a failure of HO-1 induction, it has been demonstrated that LXA_4_, restored the HO synthesis and activity, rescuing the wound healing phenotype (Biteman et al., 2007).

Overall, the therapeutic potential of SPMs in the treatment of DR are promising.

## Realizing Therapeutic Potential

The therapeutic challenges presented by diabetes-associated complications such as DAA and DKD are well documented, and experimental evidence, as outlined above, suggests a role for SPM-based mimetics as adjuvants to current therapies. Clinical trials specifically investigating the therapeutic potentials of LXs and Rvs have been limited.

In a randomized controlled trial, AT-LXA_4_ and a comparatively stable analog of LXB_4_, 15R/S-methyl-LXB_4_, reduced the severity of eczema in a study of 60 infants (Wu et al., 2013).

A synthetic analog of RvE1 is in clinical phase III testing for the treatment of the inflammation-based dry eye syndrome; along with this study, other clinical trials using an RvE1 analog to treat various conditions are underway, such as in a single study where inhaled LXA_4_ decreased LTC4-initiated bronchoprovocation in patients with asthma (Basil and Levy, 2016). RvE1, Mar1 and NPD1 are in clinical development studies for the treatment of neurodegenerative diseases and hearing loss (Serhan et al., 2015; Basil and Levy, 2016).

A clinical trial phase-I evaluating the effects of n-3 fatty acid supplementation on plasma SPMs in patients with CKD showed that endogenous production of SPMs was increased after 8-weeks n-3 fatty acid supplementation in patients with CKD, potentially impacting also patient risk of CVD complications (Mas et al., 2016).

More recently, Gilroy introduced the above mentioned first translational cantharidin-induced skin blister model in healthy male volunteers, providing insights into the mechanisms of self-resolving infections in humans, identifying cells and soluble mediators that may control the resolution phase. Further use of this model will improve our understanding of the evolution and resolution of inflammation in humans, how defects in these over-lapping pathways may contribute to the variability in disease longevity/chronicity, and lends itself to the screen of putative anti-inflammatory or pro-resolution therapies (Motwani et al., 2016).

## Summary, Conclusions, and Future Perspectives

Aging populations, increasing urbanization and widening social inequalities are all contributing factors to the rapid rise in diabetes prevalence seen over the past 40 years worldwide. Reducing premature mortality from non-communicable diseases, including diabetes, has become a global priority. For people with either T1D or T2D, advances in clinical care, such as development of better glucose-lowering drugs and structured education programs promoting life-style changes, have led to considerable increases in life expectancy. Effectively, more people are living with diabetes for longer. Understanding the disease course, onset of complications, and comorbid conditions is critical to improving specialized care for people with diabetes.

The most prevalent complications are affecting the microvascular (DKD, DR) and the macrovascular (DAA) systems. As mortality from cardiovascular complications continues to decline, attention must be turned to identifying, preventing, and treating other diabetes complications. In this context, advances in research in the molecular biology of such complications unveiled novel players and novel unified mechanisms driving different diabetes related complications. As highlighted here, inflammation is central to these processes. Evidence is accumulating that agonism of resolution of inflammation is a rational and tractable target that may be an attractive adjuvant in the context of chronic complications of diabetes applying a novel therapeutic paradigm to a vast and growing unmet need.

## Author Contributions

MdG, EB, and CG conceived and designed the review article. MdG prepared the first draft of the manuscript. CM, DA, AC, JH, EB, and CG contributed to the final version of the manuscript. All authors read and approved the final manuscript.

## Conflict of Interest Statement

The authors declare that the research was conducted in the absence of any commercial or financial relationships that could be construed as a potential conflict of interest.

## Abbreviations

18R-/17S-HEPE: 18R-/17S-hydroeicosapentaenoic acid
ABC-A/G: ATP-binding cassette-subfamily A/G
ACs: apoptotic cells
ACE: angiotensin-converting enzyme
AGE: advanced glycation end-product
AKI: acute kidney injury
ALX/FPR2: lipoxin/*N*-formyl peptide receptor-2
Apo: apolipoprotein
ATL: aspirin-triggered LX
ATLa: aspirin-triggered LX analog
BM: basement membrane
BMI: body mass index
BMP: bone morphogenetic protein
BRB: blood-retinal barrier
CAD: coronary artery disease
CANTOS: Canakinumab Anti-inflammatory Thrombosis Outcome Study
CARDS: Collaborative Atorvastatin Diabetes Study
CCR: CC-chemokine receptor
CD: cluster of differentiation
ChemR23: chemerin-like-1
CTGF: connective tissue growth factor
CVD: cardiovascular disease
DAA: diabetes-associated atherosclerosis
DAMPs: damage-associated molecular patterns
DHA: docosahexaenoic acid
DKD: diabetic kidney disease
DPP-4: di-peptidyl peptidase-4
ECs: endothelial cells
ECM: extracellular matrix
eGRF: estimated glomerular filtration rate
EMT: epithelial-mesenchymal transition
EPA: eicosapentaenoic acid
ESRD: end stage renal disease
GADA: GAD autoantibody
GLP-1-RA: glucagon-like peptide-1 receptor agonist
GPCRs: G-protein coupled receptors
HDL: high density lipoprotein
HK-2: human proximal tubular epithelial
HO: heme-oxygenase
IHD: ischemic heart disease
IKK-β: inhibitor of nuclear factor kappa-β
IRI: ischemia reperfusion injury
JAK/STAT: janus kinase/signal transducers and activators of transcription
LDL: low density lipoprotein
LDL-R: LDL-receptor
LO: lipoxygenase
LX: lipoxin
MaR: maresin
MARDs: mild age-related diabetes
M-CSF: macrophage colony-stimulating factor
MI: myocardial infarction
MMP: matrix metalloproteinase
MOD: mild obesity-related diabetes
NCCD: non-communicable chronic disease
NF-kB: nuclear factor kappa beta
NLRP, NACHT: LRR and PYD domains-containing protein
Nrf2: nuclear factor (erythroid-derived 2)-like 2
ox-/ac-LDL: oxidized-/acetylated-LDL
PD: protectin
PDGF: platelet-derived growth factor
PMNs: polymorphonuclear neutrophils
PUFA: polyunsaturated fatty acid
RAAS: renin–angiotensin–aldosterone system
RAGE: receptor for advanced glycation end-product
RCT: reverse cholesterol transport
RF: renal fibrosis
ROS: reactive oxygen species
Rv: resolvin
SAID: severe autoimmune diabetes
SFA: saturated fatty acid
SGLT-2: sodium glucose cotransporter-2
SIDD: severe insulin-deficient diabetes
SIRD: severe insulin-resistant diabetes
SMC: smooth muscle cell
SPM: specialized pro-resolving mediator
SR-A: class A-macrophage scavenger receptor
STZ: streptozotocin
T1D/T2D: type-1/type-2 diabetes
TF: tissue factor
TGF-β1: transforming growth factor-β1
Th: T helper
THBS-1: thrombospondin-1
TIF: tubulointerstitial fibrosis
TLR: toll-like receptor
VEGF: vascular endothelial growth factor
